# Machines first, humans second: on the importance of algorithmic interpretation of open chemistry data

**DOI:** 10.1186/s13321-015-0057-7

**Published:** 2015-03-22

**Authors:** Alex M Clark, Antony J Williams, Sean Ekins

**Affiliations:** Molecular Materials Informatics, 1900 St. Jacques #302, Montreal, H3J 2S1, QC Canada; Royal Society of Chemistry, 904 Tamaras Circle, Wake Forest, NC 27587 USA; Collaborations in Chemistry, 5616 Hilltop Needmore Road, Fuquay-Varina, NC 27526 USA; Collaborative Drug Discovery, 1633 Bayshore Highway, Suite 342, Burlingame, CA 94010 USA

**Keywords:** Cheminformatics, File formats, Open lab notebooks, Public data, Machine learning

## Abstract

The current rise in the use of open lab notebook techniques means that there are an increasing number of scientists who make chemical information freely and openly available to the entire community as a series of micropublications that are released shortly after the conclusion of each experiment. We propose that this trend be accompanied by a thorough examination of data sharing priorities. We argue that the most significant immediate benefactor of open data is in fact chemical algorithms, which are capable of absorbing vast quantities of data, and using it to present concise insights to working chemists, on a scale that could not be achieved by traditional publication methods. Making this goal practically achievable will require a paradigm shift in the way individual scientists translate their data into digital form, since most contemporary methods of data entry are designed for presentation to humans rather than consumption by machine learning algorithms. We discuss some of the complex issues involved in fixing current methods, as well as some of the immediate benefits that can be gained when open data is published correctly using unambiguous machine readable formats.

**Graphical Abstract:**
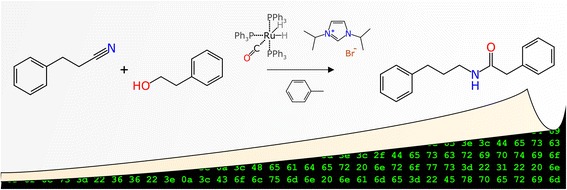
Lab notebook entries must target both visualisation by scientists and use by machine learning algorithms

Lab notebook entries must target both visualisation by scientists and use by machine learning algorithms

## Background

The increasing availability of freely accessible data for chemical compounds and their associated properties and web links is driving a significant shift in the way research is carried out. The multitude of public databases [1-6], freely distributed vendor compound libraries^a^ and directly shared lab notebooks [7] make it possible for scientists to prospectively gather together a large knowledgebase. The data may be useful to test a hypothesis in the laboratory or to build computational models. Traditionally this process involved scouring the peer reviewed literature, either online through paywalls or physically within the walls of a library, and in some cases perusing privately collected data on the subject [8]. The reuse of such data may require data licensing and we have suggested some rules that could be helpful [9].

Despite the major shift that is trending right now, there is an important caveat: many of the hosts of online data do not necessarily give proper consideration to what may well be the most important consumer of their data, namely software algorithms, especially at a time when the ongoing development of the semantic web is hyperdependent on algorithms and mappings. A scientific publication is typically downloaded and perused by hundreds or perhaps thousands of humans, but the number of people who carefully study the data content, by carefully examining the constituent chemical structures, physical properties, reaction schemes, spectral assignments, etc., is usually just a handful. The inherently low scalability of scientists’ time is in stark contrast with the ever increasing ability of software algorithms to assimilate vast quantities of data and deliver meaningful insights that could not have been observed by more traditional means. The ability for a well-designed informatics platform to productively use as much data as can be made available means that in principle every publicly available scientific data point that is relevant to a machine learning algorithm’s domain should be injected into the training set. Were this ideal state of affairs to be achieved, it would mean that every hard-won experimental result would have its chance to inform future experiments, rather than languishing in obscurity. Chemists would be able to benefit from *all* prior art within the field, and the quality of insights would improve over time as the volume of data increases and algorithms are improved.

While there have been many efforts to extract such data from the literature, there are major flaws with the methods used for extraction. The root cause is that the data entry is seldom being done by the scientists who were responsible for the experiment: for the most part, machine readable data from the published literature is created by paid curators or algorithms designed to extract information from the intractable formats used by the primary literature and patents [10]. A mistake made by a human curator, or an algorithmic extraction method, is unlikely to ever be verified by an expert familiar with the original experiment, which means that even if the provenance of the data is recorded (i.e. a citation to the original source), it is statistically improbable that it will be verified once it is incorporated into a database.

The reality of machine readable data in 2015 is that most collections of chemical structures and properties have been laundered through a number of data entry sources, few of which record the original pre-digital origin, and even fewer of which were created by scientists who are both connected with the research and have a personal vested interest in ensuring that the digitally represented version is correct. While scientists take great pains to ensure that graphical figures in their manuscripts are free of error, since it is a career-affecting embarrassment to publish incorrect data for cognition by other humans, there is no such community-enforced covenant for data that is intended for consumption by algorithms.

The disconnect between human- and machine-readable content also gets to the heart of the notion of *scalability* of scientific data. In most parts of the contemporary technology industry, software scalability refers to the ability to handle larger numbers of bytes, whether it be by ramping up database storage from gigabytes to terabytes to petabytes, or by serving millions of web page views per unit time. For the experimental sciences, the critical limitation is the evaporation of context. For example, a scientist who has been working on a project for a few weeks could have each experiment written down in shorthand notation in a paper notebook, and easily recall the remaining details from memory. After a year, shorthand notes and abbreviated sketches may be insufficient; once the lab notebooks start to pile up, other scientists start making use of the recorded processes, and eventually the original scientist moves on to another project or leaves the institution, an experimental record is seriously deficient without detailed explanation. It is all too often the case that there is insufficient context to recreate what was once institutional knowledge: the science is now effectively lost. This notion of scalability across time and personnel is a consistent entropic trend within experimental research groups, which is managed to some extent by executively curating the information that is deemed most worth preserving, and documenting it in more detail. This is formalised when preparing a manuscript for publication, or writing a thesis or research report. For releasing open data directly to the Internet, however, these mechanisms are stripped away: data that can be consumed in real time by a complete stranger on the other side of the world, or by a software algorithm, is completely dependent on whatever context was contained within the electronic document at the moment it was released. Scientific data with incomplete context can be corrected by an expert, who can infer missing information from personal knowledge, from the literature, or by conducting additional experiments in order to obtain the missing information. But these steps are the very definition of an *un*scalable process, and indeed this is the very problem that open data is attempting to solve.

Formats such as PDF files, HTML pages, word processor documents, and bitmapped or vector graphics are effectively *dead* formats, as far as machine interpretation of chemistry is concerned. There are efforts to extend the formats using chemistry enabled capabilities, examples being Chem4Word [11] but this has limited reach and capability relative to the overall needs for data access. While there has been significant success in many fields regarding the interpretation of human readable text, the obvious example being Internet search engines, the same cannot be said for chemical structures, which are a fundamental datatype in chemistry. Because chemical structures, and meta-groups of structures such as reaction schemes, are represented using opaque formats intended only for visual display, it means that almost all published chemical information is essentially dark data. Noble efforts to extract this information by text mining of chemical names [12-15] or optical structure recognition [16-18] have resulted in an error rate that is so high that it is arguably making the data scarcity problem worse. Injecting such data into the overall knowledgebase without provenance degrades the ability of any efforts to use this information. On the other hand, efforts to encourage scientists to publish quasi-formatted data, such as Excel spreadsheets, online collaborative documents, or comma separated text files with SMILES or InChI hash codes, are problematic. While these formats have a much higher degree of machine interpretability than those designed only for visual presentation, they are highly flawed due to a combination of incompleteness of description and high degrees of freedom, which join forces to ensure that such data sources are rarely meaningful to software without an expert scientist on hand to provide the missing context.

The thesis of this article is that chemical data in general, and freely available open data in particular, needs to undergo an inversion of priorities: whether explicitly or not, when scientists publish chemical information, their first and most important customer base is software algorithms, while their secondary audience is human beings familiar with the subject material. The justification for this ranking is quite simple: machines are difficult to please. They have no ability to acquire context, and whenever they are required to make a judgment call, they are only as good as the foresight of their programmer, who needs to have anticipated any possible form of ambiguity and preemptively designed a foolproof solution for resolving it. Since this is almost never completely the case for unsupervised algorithms, it is generally appropriate to assume that when handing over data with more than one possible interpretation, the algorithm will end up guessing which is correct, and frequently guessing wrong. And, to make matters worse, the results of these interpretation guesses are often stored in persistent form, which happens every time a format interconversion occurs, meaning that data that was initially flawed and incomplete becomes even more so as it is propagated. This is, in a nutshell, why most chemical data is inaccurate [19-23]. The solution to this problem is to bring the originating scientist directly into the loop, and ensure that they are involved in making sure that the data is meaningful to software, and by induction, therefore can also be made meaningful to other scientists. While much of the burden for this transformation will be dependent on greater awareness and training of the experimental scientists who create the data, the expectation of progress is only realistic if it can march in lock-step with improvement of the standard tools that chemists use for data entry, as well as improvements to the data submission standards mandated by those in charge of data collection (e.g. publishers, librarians, database curators, etc.). This parallels an increasing need, especially in academia and early career immersion, in routine procedures regarding structure representation and searching.

An effective workflow for the creation and distribution of chemical data is:

1. The scientist (who was directly involved with the research) enters the data, which typically includes structure diagrams, numbers, and other annotations;
2. The data is sent to an algorithm which attempts to parse the data, and in the event that any data has 0 or >1 possible interpretations, the problem is reported or warnings issued, and the data is rejected;
3. The scientist views the data as rendered according to the interpretation of the algorithm; once this is consistent with the scientist’s original assertion, it can be released openly, in its raw, machine-interpretable form;
4. A service can be conveniently invoked to turn the machine-friendly data into diagrams that can be viewed in a form that is most convenient to any scientist who wishes to view the data, and can be easily embedded within a common manuscript format.

We are at this point particularly concerned with chemical structure representations, their composition within larger schemes such as reactions, and their association with measurement data such as physical properties. This approach can be extended to analytical data including, where feasible, validation checking between spectra and their associated compounds [24,25], or CIF checking [26]. In the greater scheme of things, the amount of detail and nuance contained in any scientific experiment involves far more context than just molecules and corresponding data, but chemical structures and simple properties are a good place to start, since they are so fundamental: without representing these well, there is little hope for any of the remaining data. The status quo for data entry and representation of structures and properties leaves considerable room for improvement.

In the remainder of this article, we will discuss some of the existing services that are working towards this idealised workflow, some of the common pitfalls, and practical methods for working around them.

## Methods

### Public databases

**Figure 1:**
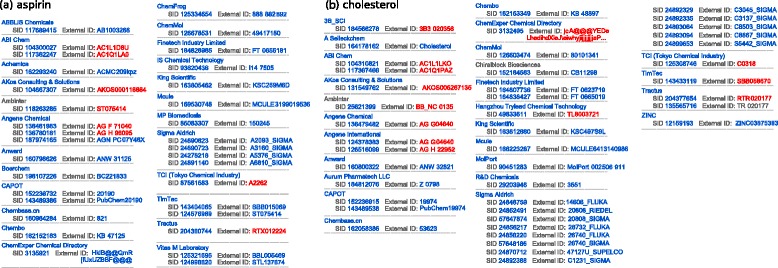
**Vendor links for PubChem records of aspirin (a) and cholesterol (b).** Links shown in red were unavailable or broken when verified (October 2014).

A number of public databases are currently available, but there are three prominent examples which represent important data quality approaches: PubChem, ChemSpider and ChEBI [27]. PubChem is the largest of the three, and like ChemSpider, it provides a method for user-driven release of chemical structures and associated data. ChEBI, on the other hand, makes use of a careful data curation process, which greatly increases the odds of an individual record being accurate, but it also keeps the data size much smaller, and passes on a significant expense to the data maintainer. PubChem and ChemSpider both aspire to be comprehensive databases, but differ in their quality strategies: PubChem relies primarily on upstream data correction, while ChemSpider is novel in that it has incorporated a significant post-upload “crowd curation” component. While both of these strategies are sound in principle, and have worked well in many other fields, they have met with modest success with regard to keeping quality high for millions of chemical compounds. In the case of PubChem, the problem is that many data depositors provide content that they have no immediate responsibility for, i.e. it had already been laundered through many error prone curation techniques and lost all provenance, and the depositor has no motivation to correct problems. Figure 1 shows a portion of the PubChem search results for aspirin and cholesterol respectively, where each of the vendor-supplied links to product pages is marked in red for unavailable links. Whether the broken links indicate that the submitting vendor is no longer in business, no longer selling the product, or has changed the identifier without resubmitting a new one, is not known. It should be noted that this issue of “link decay” is a general issue for all data aggregators.

**Figure 2:**
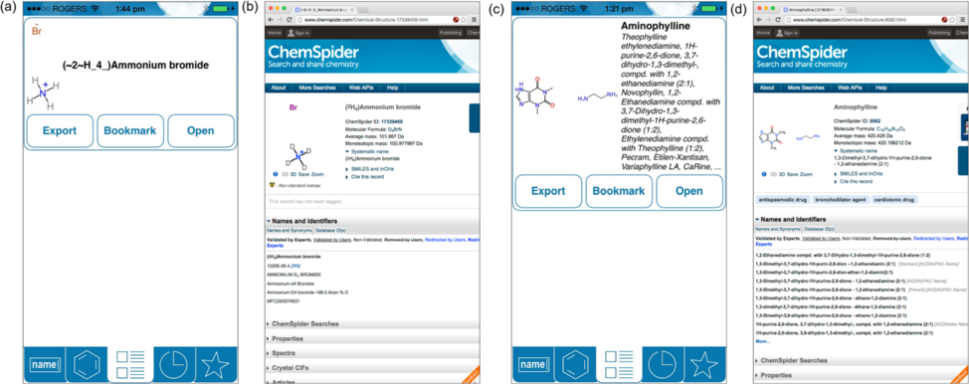
**Two ChemSpider search results: deuterated ammonium bromide, (a) and (b), and aminophylline, (c) and (d).** Examples **(a)** and **(c)** show the result as accessed by the ChemSpider Mobile app using the public API, while examples **(b)** and **(d)** show the web browser result page.

In the case of ChemSpider, many tens of thousands of erroneous data contained within large depositions have been edited out by pre-filtering routines using desktop software [28] and there are many examples where known molecules have been fixed by users of the service, but this approach runs into issues with scale: there are tens of millions of records, and the number of end users motivated to report problems numbers only in the low hundreds [29]. More troublesome, though, is that the crowd curation approach is generally only effective for spotting errors that are either obvious or popular. For example, a search for deuterated ammonium in ChemSpider reveals a problematic result: Figure 2(a) shows the result when it is discovered using the ChemSpider Mobile app [30], which acquires the structure via the public API [31], which delivers a structure representation that lacks the isotope information for the 4 deuterium atoms. Figure 2(b) shows the same result using the web browser, which renders a structure that correctly identifies the isotopes, but omits the negative charge on the bromine atom. Given the context information, most importantly the name and list of synonyms, it is quite obvious to a chemist that it is the structure that is wrong, and a solution can easily be proposed. This is an example of where crowd sourced quality improvement works quite well, because no contextual expertise is required to identify both the mistake and the correct answer, and indeed the data may well have been corrected by the time this manuscript is published.

The second example, shown in Figure 2(c) and (d) for mobile and web results respectively, shows a structural representation of a drug material: aminophylline. It is not immediately obvious that there is anything wrong with the structure, given that the active ingredient is drawn correctly, and the adduct is present. However, the synonyms that have been imported for the record are quite explicit about the adduct ratio being 2:1, which makes the structure inconsistent with the primary name and all of the synonyms. It is not necessarily clear to most chemists whether the structure should be modified, or the name, or whether the distinction is important. In this case a resolution is quite likely to be obtained, because the compound in question is a well studied drug, and there is a fair chance that an expert with specific knowledge will encounter the datum and be able to provide an authoritative correction. However there are tens of millions of compounds that are much more obscure, and any of them could have an accidental extra methylene, an incorrect chiral centre, or any number of errors that encode for molecules that are valid drawings. A structure that is valid, but represents the wrong molecule, will never be corrected by an algorithm, and will probably never be encountered by the handful of scientists who were involved in its use or synthesis. In these large majority of cases, the mechanism by which the corrupted data was injected into the greater corpus of knowledge has created a permanent stain.

### Vendor catalogs

One of the major factors behind the increasing availability of chemical structure data is the business value that is associated with a vendor of chemicals making their wares as easy as possible to find. Making it simple for anyone to incorporate structures and product identifiers into a generic chemical searching service is a clear value proposition, and an additional encouraging feature of these data sources is that a company that is responsible for selling a physical package with a particular chemical compound has a high degree of liability, and is hence motivated to make a reasonable effort to correctly represent the data.

Unfortunately these primary sources are diluted by providers of large scale high throughput screening data, who prime their customers to expect a certain degree of noise, and the potentially poor state of the informatics component is just one of the many failure modes for experiments that are designed to use quantity to compensate for lack of quality. And, perhaps more problematic, are the large number of companies that collate vendor catalogues from many other sources, losing much of the provenance along the way, and introducing layers of errors that cannot be traced back to a single source. Many of these repackaged vendor libraries have been submitted to public databases such as PubChem and ChemSpider, and many of the companies are no longer contactable, and for whom there is no business value proposition to propagate error corrections. It should be noted that the hosts of the ChemSpider database developed more stringent acceptance criteria regarding vendor catalogues soon after the initial release of the database [28]. Coupled with their pre-filtering efforts and providing direct feedback to the vendors themselves to encourage clean-up of their data has resulted in improved data quality not only in later depositions into ChemSpider but likely also for the community in general, but this is an ongoing effort.

### Open notebooks

From a data quality perspective, the most promising property of open lab notebooks is their directness. The term refers to a specific kind of electronic lab notebook that is made openly available to the scientific community shortly after the experiment writeup is complete, circumventing the usual lengthy publication cycle and any proprietary access restrictions. Typically a data unit, whether it be a reaction, a measurement, or a characterisation analysis, is prepared and released directly by the scientist who performed the experiment, and in some cases a second opinion is provided by a principal investigator or reviewer (albeit with a much faster turnaround time than for conventional publication, and also foregoing the requirement of novelty). This means that not only is it possible to find out the individual and organisation responsible for the contribution, but it also introduces the opportunity for the experimental scientist, whose knowledge at that point exceeds that of anyone else on this specific piece of data, to verify that the transmission of the data was carried out correctly.

A pertinent example is ChemSpider SyntheticPages (CSSP) [32], which is an online “micro-publishing” site serving chemists interested in chemical syntheses. Chemists are encouraged to publish the details of their experiments in order to communicate the details of their work. CSSP uses a template-based entry form and multimedia support including interactive display of various types of analytical data. The articles are reviewed by the CSSP editorial board, made up of university professors, as well as then being peer-reviewed by the incorporation of public commentary post-publication. The pre-publication peer review is generally very fast (24–48 hours) and even post-publication edits can be made as CSSP is a hybrid publication-database. Each micro-publication includes a digital object identifier (DOI) making the CSSP contribution a citable object on a CV.

It is the scientist-to-Internet transmission step that we believe is in most need of attention. Most chemists working to produce new knowledge in experimental laboratories are not trained cheminformaticians, and have a strong tendency to follow currently accepted best practices for documenting their results. At the present time, this typically involves using off-the-shelf documentation software, such as the ubiquitous Microsoft Word and Excel, and software such as ChemDraw or ChemDoodle that is specially designed to help chemists create graphics for incorporation into such general purpose packages. Unfortunately the use of these software tools all too often makes correct machine interpretation of the data impossible: even in cases where data from drawing packages is available, the reality is that these tools are designed for creation of diagrams, not machine interpretable data, and there is no guidance as to which visual aids are completely and unambiguously meaningful to an algorithm. There are documented standards for visual representation [33,34], but there has been little effort to implement these for the purpose of lossless interconversion between presentation and informatics.

Much attention of late has been given to modern online collaborative tools, such as using Google Docs to coauthor and share content, and for using electronic lab notebooks (ELNs) with a blog-like interface [35]. While excellent for sharing data in real time, they do nothing to solve the problem of machine interpretability of chemical data. Freeform text and uploading of arbitrary supporting files gives the maximum scope for scientists to describe their experiments, but it is also the worst case scenario for creating a fully automated script to gather diverse data into a single collection of relevant content in order to provide actionable intelligence. Some progress in terms of checking data formats is being made by the utilisation of chemistry specific components into the Labtrove platform [36].

**Figure 3:**
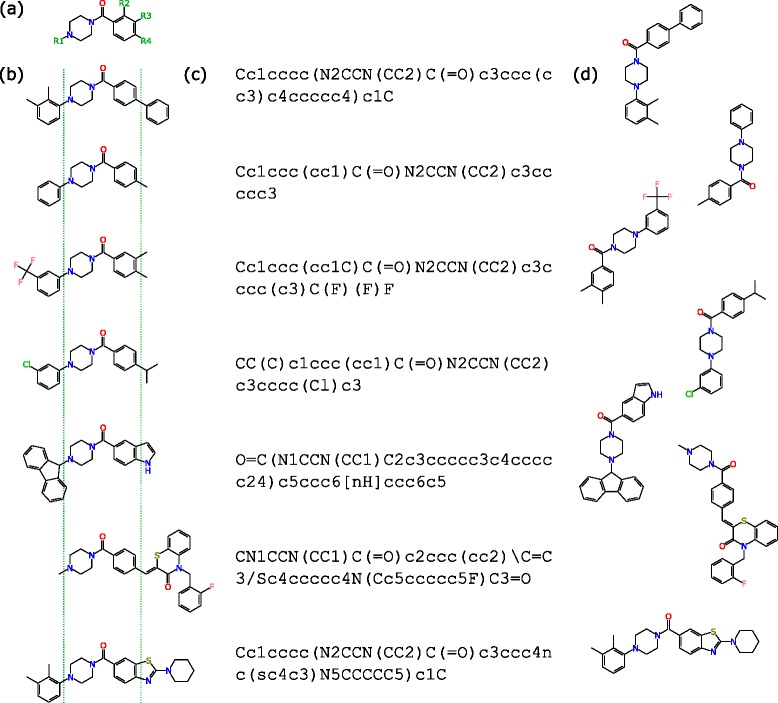
**A selection of compounds (b) based on a common scaffold (a).** Canonical SMILES strings are shown in **(c)**, and their re-depicted structures shown in **(d)**.

There are other approaches, such as disciplined use of spreadsheets for the purpose of producing comma/tab-delimited text files, where structures are represented as SMILES or InChI strings, or database identifiers. Consider the example shown in Figure 3, which shows several molecules from a series of molecules created as potential tuberculosis inhibitors, all of which are based on a common scaffold template, shown in 3(a). The structures used in the dataset, shown in 3(b), are drawn in a way that makes the common structure evident to a chemist. Were these structures to be converted into a line format, such as SMILES or InChI, in order to cram the data into a text file or spreadsheet, the nuances of layout and orientation would be lost: the re-depicted structures, shown in 3(d), are oriented randomly and do not reveal their commonality to the perceiving chemist, at least not without some careful study. For certain purposes, the structures in 3(b) and 3(d) are the same (e.g. looking up in a database or calculating properties), but for purposes of communicating the information to chemists, they are quite different when considered as a collection. Re-derivation of scaffolds and orientation can be attempted after the fact, but it is far more advisable to avoid destroying the information in the first place [37-39]. Also, changes in atom order, Kekulé/resonance form, treatment of implicit hydrogens, standardisation of functional groups or tautomers, and failure cases when using molecular bonding arrangements that are outside of the domain of the representation, means that often what should have been a commutative operation results in a structure that has many more points of difference than just layout.

The pitfalls of using line notation for structures also apply when using database references in lieu of structure, which is an option when all of the entries are known to already be in the database, e.g. using ChemSpider ID codes, but this is equally destructive of data. A public database will typically have one globally preferred structure representation which has been normalised and drawn in some preferred manner which is not necessarily most appropriate for the task at hand. Using external identifiers also introduces a slew of additional issues, e.g. if access to the Internet resource is interrupted, the data becomes unusable until it is restored. Also, records can be changed or deleted, and there is always the possibility that the database provider may one day cease to provide the service at all.

Besides issues with structures, there are many other reasons to avoid using an overly simplified text representation when properties are being included. For example, a comma delimited text file might contain a heading column described as “IC50”, and for which each following row has a number. It is pervasive practice to omit any further information such as units, errors, target, sample size, conditions, etc., which means that data stored in these files contains an enormous amount of implied context. If the data is being shared between two scientists working on the same project this may not be an issue, but if it is being uploaded into an aggregate dataset for purposes of machine learning or database reference, it is worse than useless, due to its lack of provenance and context.

### Data formatting

When releasing open chemical data in a format intended to be parsed by both humans and machines, there are many options for file format encodings, but few that meet all the necessary requirements. Data that is being associated with one or more chemical structures needs to begin by taking into consideration the ability of the selected file format to preserve all of the relevant nuances of a chemical drawing. There are many details to consider, but at the most basic level, there are two important questions that should be asked:

1. Can an algorithm correctly and unambiguously determine the molecular formula?
2. Is it possible for software to use the representation to create a diagram that reflects what the scientist originally drew?

There are surprisingly many chemical structure formats that are unable to guarantee that an algorithm can determine the correct molecular formula from the drawing. These shortcomings have mostly to do with implicit hydrogen atoms and inline abbreviations. The implicit hydrogen problem is a side effect of chemists’ shorthand, and works well in simple cases, but poorly defined valence rules for unusual bond types, and the absence of a common method for overriding the default formula, means that many nontrivial molecules cannot be drawn using the most popular cheminformatics file formats, such as MDL Molfile [40]. Abbreviations are also a persistent problem, since many structures are difficult to represent in a human-readable way without abbreviating certain groups, but since there is no universal repository for abbreviations, and many research groups invent their own overlapping sets for localised use, it is necessary to have a way to define these internally as part of the structure definition. Additional problems are introduced when using drawing software designed for diagram creation, which offers a large variety of drawing primitives that have no meaning at all (e.g. circles, symbols, free form text, etc.). These file formats are a superset of the collection of meaningful objects, and they cannot be used unless the operator has a strong understanding of which objects are valid and which are not. This information is not generally known to experimentalists, and not communicated by the drawing software.

The need to recreate a diagram with the original layout, orientation, wedge-bonds, resonance patterns and various other nuances rules out the use of any popular line-based formats that exclude atomic coordinates. There are many advocates of the use of SMILES or InChI codes for raw structure representation [41], mainly because they are convenient for storing in spreadsheets or text files, and have intrinsic canonical properties. Both of these features enable limited use of chemical data by general purpose software that has no cheminformatics capabilities, which is often a necessary evil for data manipulation. However, the amount of data destruction involved in converting a 2D sketch into a short canonical string is highly detrimental to data integrity. As long as chemically aware software is available, there are no advantages to using canonical strings to represent structures, since these can be derived on demand from the original representation, which creates a break-even-or-lose scenario: for this reason it should not be done unless there is no alternative^b^.

One of the chemical structure formats that meets the objectives of data integrity more effectively than most is that used by the *SketchEl* [42] open source chemical structure editor. The format is designed to capture 2D sketches, but takes an extreme minimalist approach to its core datastructure [43] which enumerates a list of atoms, and a list of bonds, as indicated in Table 1. Notable features are the inclusion of the zero-order bond and the option to control the number of virtual hydrogen atoms associated with each actual atom [40]. Using these simple additions, it is possible to describe any molecular species and ensure that the molecular formula is correct, regardless of how exotic its bonding arrangements are, and for the most part it is possible to devise a reasonable representation of the atom valence states.

**Table 1:** **Atom and bond properties, and currently reserved extensions, used by the** ***SketchEl*** **molecule format**

**Atom core properties**	
*Element*	An arbitrary string, which typically matches one of the symbols from the periodic table. If not an element, and there is no inline abbreviation for the atom, then the overall representation does not encode a molecule, but rather a template or query.
*x, y*	2D layout positions, in quasi-Angstrom units, with the idealised bond length being 1.5.
*Charge*	Formal atomic charge for the chemical species: must be an integer.
*Unpaired*	Number of unpaired electrons: a whole number. This is used to help calculate the valence, and is primarily relevant only for main block elements.
*Virtual hydrogens*	By default, implicit hydrogen atoms are calculated automatically for C, N, O, P and S, and zero for all other elements. Non default values allow the number of extra hydrogens to be specified explicitly, as 0 or more.
*Extensions*	An arbitrary list of strings associated with the atom, some of which have prefixes that are reserved (see below).
**Bond core properties**	
*from, to*	The two connecting atoms for the bond.
*Order*	Bond order: a whole number, which is typically one of 0, 1, 2, 3, 4 or 5. Values of 4 and 5 are extremely rare, while values of 0 are used extensively for bonding arrangements that do not follow the simple Lewis octet rule.
*Stereo type*	Flat by default, but can also be inclined or declined (so-called wedge bonds) or non-stereospecific (usually drawn as squiggly lines).
*Extensions*	An arbitrary list of strings associated with the atom, some of which have prefixes that are reserved (see below).
**Atom reserved extension properties**	
*z*	Optional third dimension: the existence of z-coordinates implies that the molecule is not a flat 2D depiction but rather a 3D conformation.
*Isotope*	Specific isotope enrichment, where the default value of 0 implies a natural isotope distribution.
*Mapping number*	Integer mapping number associated with the atom. This can be used for any purpose, but is often for correlating atoms in a series or a reaction.
*Query*	Query properties used to specify how to match a variety of atom types.
*Abbreviation*	Inline abbreviation, containing a terminal substructure fragment that defines the entire molecular species that the placeholder atom represents. Can be recursive, i.e. the abbreviation can contain its own abbreviations.
**Bond reserved extension properties**	
*Query*	Query properties used to specify how to match a variety of bond types.

In spite of its minimalism, the format is also extensible in a way that is both forward- and backward-compatible. A number of properties are optional: these, properties that will be defined in the future, and custom properties that are not a part of the formal specification, are stored in a way that preserves the read/modify/write integrity for algorithms that do not care to implement them. This is in contrast to formats like MDL Molfile: if a software package writes a molecule that makes use of a property that is not part of the lowest common denominator subset that most implementations can handle, or defines its own extensions, the extra data will be either deleted or corrupted if it is submitted to a software algorithm that does not implement the property correctly.

**Figure 4:**
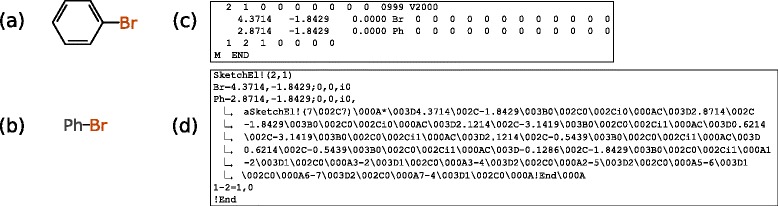
**Bromobenzene, drawn in full (a) and with an abbreviation (b).** The Molfile with the plain text abbreviation is shown in **(c)**, while the SketchEl representation with the abbreviation encoded inline is shown in **(d)**.

One of the optional extensions that can be implemented is the inclusion of inline abbreviations. For most cheminformatics formats, if the user wishes to represent a common abbreviation, e.g. the use of **Ph** to represent *phenyl*, the atom name is simply given the text “Ph” instead of a valid element. If all goes well, the cheminformatics software that is interpreting the structure will have its own special lookup list which recognises this shorthand notation. This approach is haphazard at best, and the *SketchEl* format solves the problem by defining abbreviations inline. Figure 4 shows two approaches to representing bromobenzene: in (a) the 7 heavy atoms are drawn out in full, while in (b) the phenyl (C_6_H_5_) fragment is written as a single node with a text abbreviation. If the abbreviation atom is created in the same way as for an ordinary element symbol, saving the structure as an MDL Molfile would be equivalent to the text shown in (c). (It should be noted that while the MDL Molfile specification does include a specification referred to as “S-groups” which can be used to achieve a similar effect as inline abbreviations, this file format suffers from the fact that it has literally thousands of implementations in current use across the industry, and that most of these implementations only make use of a small lowest common denominator subset. Because the format is defined in a way that makes the the load/modify/save workflow destructive of unsupported properties, any property that is outside of the commonly implemented subset is frequently at risk of being deleted or corrupted. Making use of features such as S-groups substantially reduces the number of software packages that are compatible with the data). This encodes for a 2-node structure, of which just one is an element symbol, and so the chemical meaning would be invalid to any parser that did not implement special meaning for the “Ph” symbol. A well specified *SketchEl* file is shown in (d), which uses the inline abbreviation form to explicitly include the fragment to which “Ph” corresponds, i.e. the phenyl ring itself, and the attachment point. The sub-fragment definition contains all of the information necessary to display or recreate the fragment, and hence represent the structure with all of its atoms, and infer the correct overall molecular formula.

**Figure 5:**
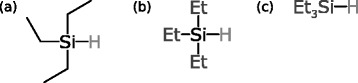
**Three different representations of triethylsilane, using different degrees of abbreviation.**

The name of the abbreviation is defined in lieu of the element symbol, and the structural fragment is defined completely within the extension field. Software that does not implement the abbreviation mechanism can read, view, modify and write *SketchEl* molecules without destroying information, while abbreviation-aware software can provide value added functionality, such as optionally showing the full structural definition by request, or providing convenient ways to select from currently available abbreviations, or subsuming existing structural fragments as new abbreviations or molecular formulae (e.g. Ph could also be represented as C_6_H_5_). Abbreviations can also be nested, e.g. a fragment defined as Et_3_Si may expand out initially to a silicon atom with 3 ethyl “atoms”, which are themselves abbreviations for C_2_H_5_. Figure 5 illustrates the levels of abbreviation: (a) is drawn out in full, with each heavy atom represented as its own node, encoding for the molecular formula of C_6_H_16_Si; (b) collapses each of the ethyl groups into a single abbreviated composite fragment, each node of which has the implied molecular formula C_2_H_5_; (c) represents the whole silyl fragment as an abbreviation-of-abbreviations, compiling the 3 ethyl groups and the silicon atom from (b) into a single meta-abbreviation.

While the *SketchEl* format cannot intrinsically represent many higher orders of metadata, e.g. mixtures of compounds with different stereochemistry or constitutional isomers, these definitions are difficult to pinpoint with a single proto-structure in a way that is not ad hoc: a more rigorous approach is to define a higher layer of abstraction, which either enumerates the different molecular species explicitly, or in complex but regular cases such as Markush collections, defines its own enumeration formula.

Assembling collections of molecular structures brings a similar capability wish list: a format should be minimalistic, simple to define, easy to implement, and most importantly it should be forward and backward compatible, so any implementation of the specification can read, view, modify and write the data with reasonable assurance that important content will not be destroyed or corrupted. Since the most common use case scenario for multiple structures and data involves representation in a tabular format, roughly analogous to a spreadsheet with a molecular datatype, it makes sense to define the core data format in this way, whereby more exotic data arrangements are mapped onto the table with higher order metadata.

These characteristics are implemented by the datasheet XML format, which is also used by the *SketchEl* package, for editing collections of structures and data. At its core it is a very simple tabular format, where each column is strongly typed, and is one of *molecule*, *string*, *integer*, *real number,* or *boolean*. Molecules are embedded using the *SketchEl* molecule format. For many data collections, e.g. lists of molecular structures with their associated identifiers (name, link, database ID, etc.) and properties (activity, solubility, melting point, etc.), the core format is quite adequate.

For additional labelling, or for higher order organisation such as arranging multiple structures into reaction schemes, the datasheet header allows for extensions, which contain arbitrary content that is generally not shown to the end user. Extensions that follow a specific protocol are described as *aspects*. The principle of the aspect extension mechanism is that if a software application implements the aspect, it should provide additional capabilities, such as alternate viewing modes, specialised editors or additional classification information. An aspect is required to be as tolerant as possible of disruptive external modifications, e.g. if one of its necessary columns is deleted, it will recreate the column using default values. If the software application does not recognise the aspect, it should still be able to load the datasheet, present it to the user in its default tabular form, allow cells to be edited, rows to be added or deleted or moved, and in some cases modification of column names and types, without necessarily disrupting the higher order markup. For example, if an aspect defines the default units for a given column, loading the datasheet with an unaware editor and modifying the quantity values preserves the read, view, modify, save integrity, as long as the user is aware of what the numbers represent. If an aspect defines a chemical reaction, where a number of molecule columns are used to define the various components, it is possible to use a minimal editor to change some of the molecular structures, and still preserve the reaction definition.

**Figure 6:**
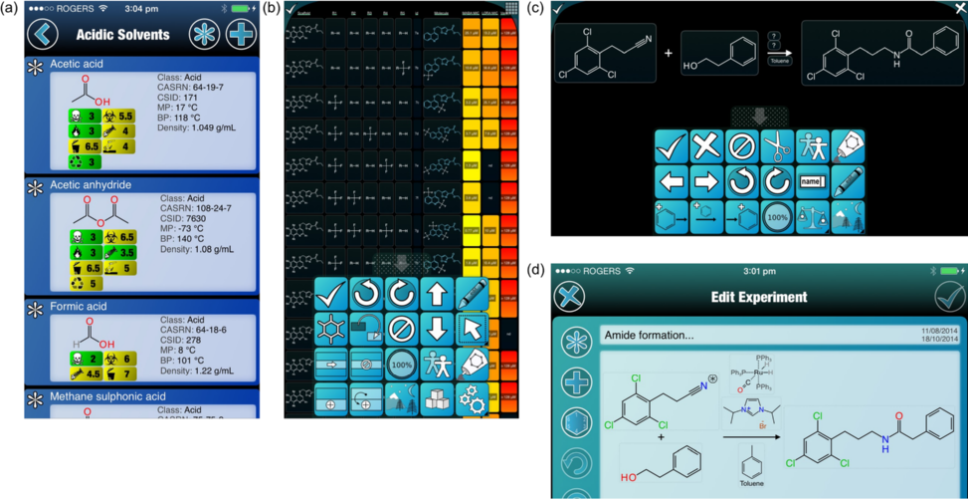
**Some of the datasheet aspects currently in use: (a)** ***Solvent*** **aspect (displayed by the Green Lab Notebook app), (b)** ***SARTable*** **aspect (displayed by the SAR Table app), (c)** ***Reaction*** **aspect (displayed by the Mobile Molecular DataSheet app) and (d)** ***Experiment*** **aspect (displayed by the Green Lab Notebook app).**

**Figure 7:**
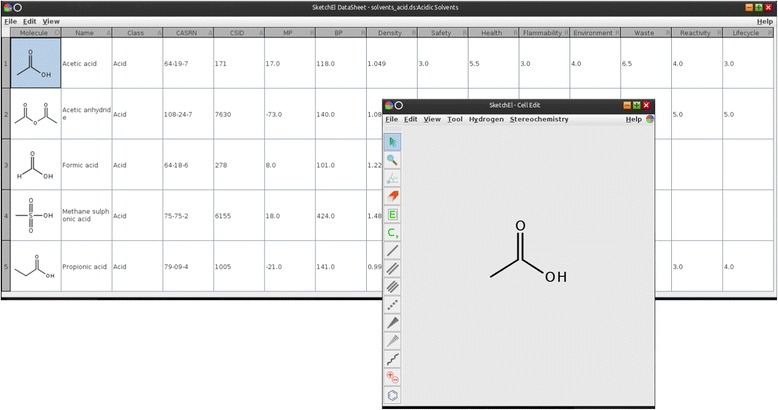
**Using the open source** ***SketchEl*** **editor to view and modify a datasheet that has an embedded** ***Solvent*** **aspect, which can be done safely and conveniently even though the** ***SketchEl*** **application does not implement the aspect.**

Figure 6 shows several examples of datasheet aspects that are currently in use. Figure 6(a) demonstrates the *Solvent* aspect, rendered using the Green Lab Notebook app [44]. The aspect definition is quite minimal, and merely suggests more visually informative ways to display several environmental and physical properties. Molecular datasheets containing this aspect can be effectively viewed and edited with a tabular spreadsheet-like editor that does not implement the aspect, which is shown in Figure 7. Figure 6(b) shows an example of the *SARTable* aspect, as rendered by the eponymous SAR Table app [45]. This aspect definition adds explicit definitions to distinguish columns of *scaffolds* and *substituents*, and the composite *molecule* that is formed by grafting them together, as well as additional information about properties regarding their units and operating range. As with the *Solvent* aspect, an unaware molecular datasheet editor can be used to effectively view and modify such data, as long as the operator takes some care to respect the column layout, but will not benefit from some powerful editing capabilities, such as scaffold detection or automatic recomposition of the molecule each time the fragments are changed.

Figure 6(c) and (d) both show renditions of aspects that encode for chemical reactions. The *Reaction* aspect, shown in (c) rendered by the Mobile Molecular DataSheet app [46], combines a number of columns containing molecules, text and numbers into a single reaction step, which is ideally rendered as a single graphical object representing multiple components. The *Experiment* aspect, shown in (d) rendered by the Green Lab Notebook app, is a subclass of *Reaction*, which augments the definitions with additional information such as quantities and component roles, and also allows for multiple steps. While datasheets containing the *Reaction* or *Experiment* aspects can be viewed and edited by software that does not recognise the aspects, the meaning of the content is less clear to the viewer, since a spreadsheet-like representation is quite different to the reaction component layout algorithms used by the software that implements these aspects.

Chemical data that is stored using the datasheet XML format, with embedded *SketchEl* molecules, conforms to a very well defined, easy to implement core specification for data purity. Both molecules and datasheets can be extended as necessary to describe more complex concepts, which are often necessary to ensure machine readability, but their core definitions are highly functional, and generally safe to edit without specific knowledge of higher order markup.

### Data entry

One of the valuable properties of open data is the close connection between the scientist and the content, which can be treated as an opportunity to solve some of the most pernicious data quality issues in chemistry. When it comes to aggregated collections of molecular structures, there are two main kinds of problems: structure representations that are demonstrably wrong in the absolute sense, and those which could be correct, but in the given context, the wrong chemical species is being described.

A significant amount of work has been invested in the former category, for example the ChemSpider Validation and Standardization Platform (CVSP) [47] (also see: Karapetyan K, Williams AJ, Batchelor C, Sharpe D, Tkachenko V: The Chemical Validation and Standardization Platform (CVSP). Large-scale automated validation of chemical structure datasets, accepted for publication to Journal of Cheminformatics). This tool embodies chemical knowledge that can search for a number of common structure mistakes, or representations that do not follow a protocol, such as covalently bound salts (Na-Cl) or pentavalent nitro groups. Many of these examples are common and easy to fix, but there are many more examples that cannot be corrected without knowledge of context. Furthermore, there is no way to ascertain whether a molecule is the right one when there are multiple reference points. For example, a data record that provides a bioactivity measurement for a molecule named “aspirin”, for which the structure given is salicylic acid, even a smart algorithm that is able to find out that aspirin is the acetylated form cannot know whether the data record provided the wrong structure or the wrong name, unless the provenance is somehow recorded. Whether the molecule or the name should be trusted preferentially, and if there are conflicts within either of these, which source has precedence, means that each data collection needs a complex and elaborate policy for judging data quality. These challenges have directly influenced some of the approaches associated with the Open PHACTS semantic web project [48] where a “chemical lenses” approach has been utilised to focus the user in on various forms of the chemical [49].

**Figure 8:**
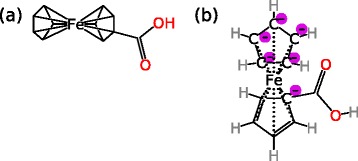
**(a) Original drawing of ferrocene carboxylic acid using a limited alphabet of bond types (CASRN 1271-42-7); (b) modified structure after automated processing (PubChem ID 11986122).**

Proactive “fixing” of structures is capable of doing more harm than good. Consider the ferrocene derivative shown in Figure 8: this compound has been imported into PubChem from several sources, one of them being the NIST WebBook [50]. The structure representation used by the NIST source is shown in 8(a): it is an admirable attempt to work within the constraints of the commonly implemented subset of the MDL Molfile format, using only simple bonding types. While the rendition fails to capture the aromaticity of the cyclopentadienyl ligands, or the oxidation state of the metal, it successfully represents all of the points of connection between the metal and the organometallic fragments, and does so in such a way that the valence states of the carbon atoms add up to the correct number, which means that even the most trivial implicit hydrogen counting algorithm will infer the correct molecular formula. Consider, however, the post-processed structure that is incorporated into PubChem, shown in Figure 8(b), which is obviously broken. The PubChem molecular structure representation has a larger alphabet of bond types than that which is in common use by most software, which can in principle be used to good effect, but in practice this is not necessarily the case. As can be seen, the processed form adds 6 negative charges to a molecule that was (and should be) overall neutral, and produces a seemingly arbitrary single/double bond pattern within the 5-membered rings. While it may seem unfair to use an example of a molecule that is difficult to represent using fundamental cheminformatics primitives, the point is that using an automated structure correction algorithm is not a same-or-better proposition: such algorithms can and do break structures that were originally correct.

Nonetheless, the tools for ensuring that a molecule is both valid and standardised according to a set of rules is extremely valuable when incorporated into the editing workflow, i.e. at the source of entry when user intervention is still an option, rather than using automated scripts further downstream. Violations can be highlighted during the editing of a molecular structure, and flagged again if the user attempts to submit the entry to a database.

The adherence to hard rules for structure validity is often appropriate for processing large databases with preexisting quality issues, but a heavy-handed approach is not appropriate for original data entry. For example, when a chemist draws a pentavalent carbon atom, it is usually a mistake, and software that can call attention to this likely mistake as early as possible is beneficial. Nonetheless, there are real reasons for representing such species (e.g. carboranes [51]), which raises an important point that is sometimes lost in software: the originating scientist is *always* the final arbiter. There is usually at that time nobody in the world who knows more about the particular unit of research being described, and the rule set designed for a particular software package is far down the list of contenders.

**Figure 9:**
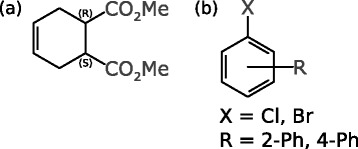
**Two descriptions of organic compounds that are unlikely to be understood by cheminformatics algorithms: (a) plain text annotation of chiral centers; (b) mixture of compounds with varied connectivity.**

Besides calling attention to what appear to be actual problems with structures, the advice that an editing tool should be providing is real time feedback on how the structure would be interpreted by an algorithm in its current state. The number of drawing conventions in common use by chemists, which are enabled by many chemical drawing packages, but have no broadly accepted cheminformatics interpretation, is enormous. Examples can be found throughout all kinds of synthetic chemistry literature. Figure 9 shows two common examples with simple organic compounds: in (a) the chiral centers are denoted using the (R) and (S) labels, which chemists often draw using plain text labels when preparing manuscripts. These annotations are meaningless to machine algorithms, and so the structure would be interpreted as having two unresolved chiral centers, which is not what is being represented. In (b), a common shortcut for pictures is used: the X and R abbreviations together encode for a total of 4 different chemical species. Even if the cheminformatics software were able to ascertain that the non-element labels are placeholders for fragments, the way in which the *ortho*/*para* isomerism is drawn makes the direct interpretation of the template a mixture of two separate connected components - C_6_H_5_X and CH_3_R - which happen to have an overlapping bond. An algorithm would have to not only use text mining to find out the composition of the fragments, but also further interpret the label and other contextual clues in order to determine that the intended connectivity is quite different to what is literally stated.

**Figure 10:**
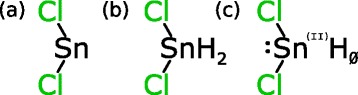
**Tin (II) chloride, (a) drawn naively; (b) interpreted incorrectly; (c) redundantly over-specified.**

While such underappreciated problems with organic compounds are prevalent throughout the literature, the problems become far worse when venturing into inorganic and organometallic chemistry. One of the archetypical examples of the failures of cheminformatics software is the case of tin (II) chloride. Most chemists would expect to be able to draw the structure shown in Figure 10(a), and to be able to rest assured that any software algorithm would understand that this represents a species with the molecular formula of SnCl_2_. There is, however, a very high likelihood that an interpretation algorithm will apply valence rules to calculate the number of implied hydrogens and, knowing that tin is in group 14, treat it the same way as for silicon or carbon, which means that the perceived structure is as shown in Figure 10(b): H_2_SnCl_2_. This is most likely incorrect, but the hydrogenated form is still a legitimate structure, albeit difficult to handle in the laboratory. One might be tempted to solve the problem by not automatically adding hydrogen atoms to tin, but this runs the risk of breaking interoperability with other software. For example, if the user had drawn methyl groups instead of chlorine atoms, the most probable structure that was being referred to is (CH_3_)_2_SnH_2_, i.e. the organotin (IV) compound, for which implicit hydrogens *should* be added. This is a pertinent example, because for tin halides, the divalent forms tend to be stable compounds, and the hydrogen-saturated form is generally highly reactive, whereas this pattern is reversed for organotin analogs, for which carbene equivalents are usually only observed as fleeting intermediates. This kind of chemical knowledge that can often be assumed of human chemists is almost never taken into account by cheminformatics algorithms, and nor should it be, given that the scope for complexity is effectively infinite. As with all such ambiguities, the solution is to properly define the rules, and include ways to explicitly indicate any exceptions. Figure 10(c) uses 3 different ways to solve the ambiguity for tin (II) chloride: indicating that there are 2 valence electrons not used for bonding; specifying the oxidation state; and explicitly marking the number of virtual hydrogens as being zero. Any one of these methods will suffice in this example.

**Figure 11:**
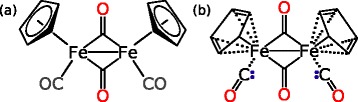
**Two representations of cyclopentadienyldicarbonyliron dimer: (a) diagram style preferred by chemists; (b) a more fundamental representation that does not mislead cheminformatics algorithms.**

Figure 11 shows a structure that is difficult to draw by any definition: an iron dimer of a commonly used synthetic fragment. The drawing in 11(a) shows a chemist-friendly graphical representation, which is suitable for publication in manuscripts [52]. As drawn with most chemical structure drawing tools, this has a number of problems. The cyclopentadienyl ring is drawn with a ring, which is a graphical object that has no meaning to cheminformatics, so rather than defining the fragment as C_5_H_5_ (with an extra electron), it is literally interpreted as cyclopentane, i.e. C_5_H_10_. The line emanating from the metal to the center of the ring is interpreted as a methyl ligand, rather than an η^5^ multicentre attachment. The use of the wedge bond to denote a 3D effect does in this case conflict with its use in cheminformatics to denote chirality, and may be stripped out or used in some way to label a chiral center that is not real. The terminal carbonyl ligands are encoded as “CO” and “OC” respectively, which are not elements, and so are meaningless. All of these issues are quite unimportant for human-to-human communication, since the mapping between these drawing conventions and the corresponding claim about the chemical reality are well understood by organometallic chemists, but the absence of a corresponding mapping from graphics-to-cheminformatics means that these diagrams are unsuitable for use by machines. Figure 11(b) shows an alternative representation of the same structure, which is restricted to use of a graph containing only atom nodes and bond edges. The use of zero-order (dotted) bonds means that the atoms are all represented with a reasonable valence count, all atom-to-atom connections have an explicit bond edge, and that the structure distinguishes between *dative* and *anionic* ligand types. The atypical bonding of the terminal carbonyl ligands is represented by explicitly indicating a carbene-like lone pair on the ligand atom, which is one way to ensure that implicit hydrogens are not erroneously added.

While the second structure diagram in Figure 11 is much less appealing for purposes of manuscript preparation, this illustrates the primary argument of this article: imagine two algorithms, one designed to automatically convert from the human-friendly format (a) to the machine-friendly format (b), and the other to perform the opposite. If both algorithms have a comparably high but imperfect success rate for a given domain (e.g. 99%), it is overwhelmingly preferable to use the machine-friendly format for the primary repository, because of the asymmetry of consequences. When a structure drawn for humans is parsed incorrectly into a machine format and injected into a database, all too often the error goes unnoticed, and if the provenance is not retained, then the corrupted data will surely find its way into the body of scientific knowledge and continue on to befoul any and all data processing operations that it comes into contact with. If on the other hand the data is represented in a machine-friendly way, and algorithmically converted into a human-friendly graphical format as needed, the consequences of failure are minor. For high quality uses such as manuscript preparation, rare flaws will generally be noticed and can be corrected easily enough, since literature publications are carefully scrutinised by several reviewers prior to publication. Even if a sub-optimal drawing is published, as long as it is correct, the fallout is likely to be manageable. For low quality uses, like browsing search results from a database query, occasional representation of structures in a way that is correct but not aesthetically ideal is a small nuisance, compared to data corruption.

As well as such valence issues, a large category of data entry issues arise from the use of text. As a general rule, any text in a structure diagram that does not map to an element in the periodic table brings with it an additional burden for ensuring that its meaning is strictly defined. Free text, e.g. a label that says “chiral” or “cis/trans”, is clearly not applicable, but as mentioned earlier, abbreviations can be dealt with by ensuring that they are defined within the chemical structure - though not with free text labels such as “L = PPh_3_”. Other kinds of abbreviations, such as X, R, R1, R2, etc., serve as element placeholders and their presence implies that the representation is of a template, rather than a structure. It is important to ensure that these structures are never submitted to a database without some accompanying formula that specifies how the template should be converted into actual chemical species, but fortunately this is easy for data curation algorithms to detect, and reject due to missing information.

One of the best visual aids for educating scientists about what their structure diagrams actually mean to a machine algorithm is simply to display the computed molecular formula for particular fragments. This is a concept that is deeply ingrained for all chemists, regardless of their level of affinity for cheminformatics software. If the example in Figure 11(a) is reported as having a formula of C_14_H_28_Fe_2_[*CO*][*OC*], the chemist does not need to be convinced that there is a problem: this is *not* the chemical composition of the molecule, which means that knowingly submitting this representation to a database is tantamount to scientific fraud, and therefore something must be done about it.

The most effective way to ensure that structures are represented accurately is to use data entry tools that operate on a fundamental datastructure, such as the *SketchEl* molecule format, or an enhanced variant of the industry standard MDL Molfile [40]. Using graphical diagram drawing tools is problematic, because the functionality they provide is a superset of what is valid for cheminformatics purposes, and there are no algorithms that can transform an aesthetically styled structure into a machine readable valid equivalent with a success rate that is acceptable. In principle, though, it may be effective to create a plugin for such software to show the machine interpretation and structural formula breakdown, updated in real time, in order to ensure that users are aware of when their stylistic choices result in misleading content, but such tools are not currently available. This represents a potential unmet need.

### Case studies

#### Direct data hosting

There are many services that store user-provided chemical data in a fundamental cheminformatics format, including the aforementioned ChemSpider and PubChem databases. These services make use of elaborate content aggregation features, which involves a large amount of automated correction. For most organic structures that conform to simple Lewis octet rules this can be trusted to leave a well-drawn structure unmolested, but problems arise when leaving this domain.

The *molsync.com* site [53] provides an example of a service that openly hosts chemical data, in its most pure form, and allows it to be consumed in a variety of different downstream formats, for either humans or machines. We describe some of the properties of this service, because it differs from most ad hoc Internet sharing facilities in that it provides interpretation *and* visualisation of raw chemical data. It demonstrates several key proof of concept features that should be standard for chemical data hosting, and can be incorporated into open lab notebook software.

Data can be uploaded using a simple REST-based API, after which point it is stored in a database and assigned an identifier. The data is typically uploaded as either a *SketchEl* molecule or datasheet XML document, but other related formats such as MDL Molfiles will be automatically converted. From the identifier, a URL can be constructed, which allows anyone with an Internet connection to be open the page in a browser.

**Figure 12:**
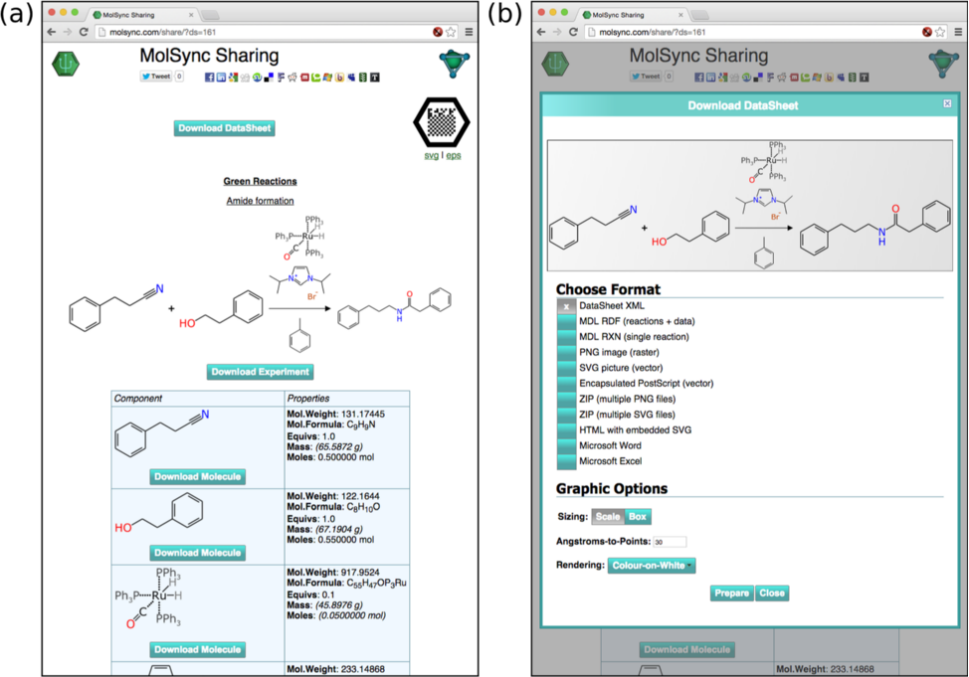
**Sharing chemical data using the** ***molsync.com*** **service, which stores the raw datasheet with any applicable aspects.** The default **(a)** view is an HTML5 page, using resizable vector graphics, which can be downloaded in a variety of informatics or customised graphics formats **(b)**.

In browser mode, a standard HTML5 page is created. The web service fetches the underlying raw data, and creates the tabular structure shown to the user, as shown in Figure 12(a).

The outline of the browser presentation uses simple HTML and CSS. Individual molecular structures, and some of the higher order metadata specified by aspects that are implemented by the server - such as chemical reactions - are drawn using a high grade rendering layout algorithm [54] and passed to the front end as vector drawing instructions, which means that the page can be rendered to any resolution, and also can be sent to a printer or converted into a PDF file without any loss of quality.

### Data exporting

There are two main use cases for data conversion: migrating to a different cheminformatics format to be consumed by a specific software application, and the generation of graphics for presentation or publication purposes.

When raw data is stored in a rigorously minimalistic and unambiguous format, it is generally effective to convert this data into the lowest common denominator subset of a less rigorous format, with some potential for information loss, which may remain theoretical for a reasonable domain of use cases. For example, converting a structure into a V2000 MDL Molfile that is readable by the large majority of software that can parse the format can be expected to preserve all of the pertinent information in many molecule types. For nonorganic structures that cannot be properly represented with the V2000 format, or for structures that use inline abbreviations, the conversion cannot survive a round trip intact, and so the conversion is an irreversible *downstream* one. For information that is pertinent to the destination format, but does not exist in the core specification of a *SketchEl* molecule, the extensibility mechanism holds the door open for future improvement, in a backward and forward compatible way. For example, MDL Molfiles provide a number of capabilities for specifying chemical queries [55] as atom and bond annotations. The *SketchEl* molecule format can optionally incorporate analogous extensions, and if the data hosting service is subsequently upgraded so that it can convert the overlapping subset of functionality to the MDL Molfile equivalent, then this capability can be introduced at any time. The operation is commutative to the extent that the definitions match.

Similarly with collections being exported as MDL SDfiles, a significant amount of metadata is lost, particularly regarding the columns and types, and so it cannot always be assumed that an *upstream* conversion will preserve all of the original data. Other destination formats have more interesting caveats. For example, the Chemical Markup Language (CML) [56] is for all practical purposes a superset of all possible chemical formats, since additional tags can be introduced by any writer without affecting validity, which passes the interpretation problem down the line: there is no guarantee that other software will understand the choice of properties, meaning that interoperability is very low.

Converting a rigorous, minimalistic cheminformatics format into manuscript quality graphics is not a simple task. Because high level aesthetic style information has no place being stored in the core definition of a datastructure that is intended to describe the chemistry in a way that is understandable to machines, it means that the rendering process involves the creation of a lot of additional information, namely the positioning for each of the labels, bonds and various other annotations [54]. While the loss of layout cues in the core datastructure is unfortunate in the case of structures that were originally imported from a drawing program that allowed the user to specify such preferences, it does mean that all structures are created equal as far as visualisation is concerned, as long as the 2D coordinates and wedge bonds for each of the non-virtual atoms are chosen to suit. Since many structures are partially or completely composed using algorithms, rather than being hand drawn, it is highly beneficial to be able to create high quality diagrams without additional user intervention. One alternative to insisting on algorithmic recreation of aesthetic properties is to store layout hints (e.g. atom colours, charge positions, etc.) as optional non-fundamental extension properties.

As with cheminformatics formats, there are a number of graphics formats to choose from, and the most appropriate of these varies depending on the destination. The most universally recognised format is the Portable Network Graphics (PNG) format, which is a bitmapped format. Until recently this was the only practical method for displaying custom graphics on a web page, but has major limitations, e.g. the resolution has to be selected prior to generating the page, as well as a litany of other inconveniences. All too often manuscripts created with wordprocessing software incorporate bitmapped graphics, and these need to be generated at a much higher resolution than what is suitable for screen viewer. A document with screen-resolution bitmapped graphics appears shoddy when zoomed to a non-default resolution, and frequently almost illegible when printed or converted into a PDF file, which describes both of the primary use cases for manuscript preparation. Since molecular structures are inherently vector diagrams, being originally composed by the software using a small dictionary of shapes: lines, circles, curves, etc., it is strongly preferable to represent the drawings in a vector format, which ensures that they can be rendered as perfectly as the device allows, whether it be a screen, a printer, or a print-ready file format like PDF. There are a number of vector graphics formats to choose from, and these include Scalable Vector Graphics (SVG), Encapsulated PostScript (EPS), and embedded graphics formats like DrawingML, which can be used to compose vector diagrams inside Microsoft Word, Excel or PowerPoint documents.

The reason for taking the approach of storing chemical data in the most rigorous cheminformatics format, and converting it on demand, is that functionality can be provided as it is needed. Because the data is stored in a format that is understandable to an algorithm at a fundamental level, it can be converted into any format that the service is currently capable of creating, and taking into account the needs of any aspects that have currently been implemented by the service. Figure 12(b) shows the dialog that is presented when requesting the downloading of a datasheet with an embedded *Experiment* aspect. The list of available formats includes several informatics formats, and number of different ways to render the content as graphics which can subsequently be used by other presentation packages, including Microsoft Word format with embedded vector diagrams. The combination of machine-readable raw data and a chemistry aware service has two clear advantages over storing pre-prepared files in several formats: additional output formats can be added at any time, and the user is also given the option to customise the output, e.g. by selecting the resolution and colour scheme for molecular graphics. This approach satisfies the needs of machines *and* humans.

### Sharing

The Internet provides a seemingly limitless menu of ways to share information across the globe, and most of them can be adapted to chemistry in some way, but other than approaches such as that taken by *molsync.com*, these seldom have the ability to form a strong association between the machine interpretable data and the human viewable rendering thereof. For example, a user can easily use Twitter to share a graphical picture of a molecular structure, but since this is just a bitmapped image, to a machine it is largely indistinguishable from a photograph of a kitten. The data only regains its full value if an individual human redraws the structure using a chemical drawing package (or attempts to parse it with a bitmap-to-structure conversion tool).

**Figure 13:**
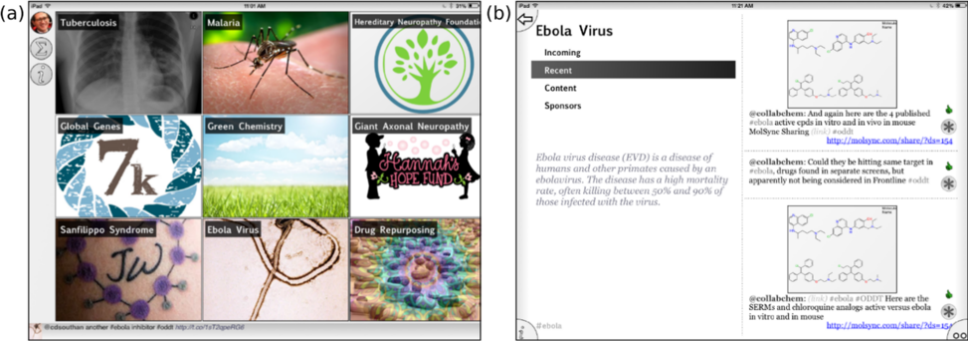
**The Open Drug Discovery Teams app showing some of the covered topics (a) and a detail view of some of the content obtained relating to the Ebola virus, in particular several structures of FDA approved drugs (b).**

On the other hand, if a user posts chemical data using *molsync.com*, or links to a verified entry in a database like ChemSpider or PubChem, or any other link that can be resolved to a download that advertises a chemical MIME type [57] it may well be crawled by information extraction services. For example, the Open Drug Discovery Teams (ODDT) project [58] continuously scours Twitter feeds looking for specific hashtags that relate to its collection of topics, which mostly pertain to rare and neglected diseases, and other precompetitive scientific topics (e.g. green chemistry and drug repurposing). Tweets with links are harvested and added to a database, but those which have resolvable chemical data are treated specially, as shown in Figure 13.

Sharing of machine interpretable data is leveraged from within the ODDT app, and it is easy to obtain it and incorporate it within a cheminformatics workflow. The data is acquired in its pure state, and there is no need to reenter it, because no information was lost during the transition. We have described how mobile technologies can be used for secure sharing of data prior to open sharing in ODDT [59]. In addition we have shown how ODDT can be used to surface structure activity relationship (SAR) data from behind paywalls [60] and raise awareness of specific topics [61,62]. Twitter is also a valuable tool for realtime microblogging from scientific conferences [63]: there are an increasing number of scientists who routinely “live tweet” what they learn during conferences, and there is no reason why digitally accessible data cannot be incorporated into this stream.

Another novelty feature that the *molsync.com* service provides is the display of a molecular glyph, which is the equivalent of a chemical QR code: its role is equivalent to a URL, except that when it is printed out on paper, e.g. on a poster or a label, it is possible to use the Living Molecules mobile app [64] to photograph the glyph. Once the payload is extracted, the app is able to go directly to the source of the data, and download it in its pure form, i.e. it is now loaded into the app itself, and from there it can be viewed, exported, re-shared or used in any other way that raw cheminformatics data can. We have shown how this glyph could be used practically to encode chemical ingredients in consumer products [65].

## Conclusion

The increasing importance of data-intensive cheminformatics algorithms, the growing recognition of problems with existing data collections, and the rising prominence of open lab notebook data means that the community has an opportunity to correct some of the persistent data quality problems that have plagued the field ever since large datasets began to be made publicly available on the Internet. Addressing these problems will require a significant amount of effort from all participants, starting with the creators of chemical software tools used for data entry. Alongside the improvement of available user-facing tools, an increased awareness is required of individual experimentalists who provide the raw data, and the cheminformaticians who build systems for collecting and assimilating it. Some of the data entry tools in current use can already be used to generate high quality machine readable data, but in many cases only if there is a significant educational push to ensure that scientists use them correctly, and this is unlikely to happen in isolation, unless the tools themselves are greatly improved. Software creators need to ensure that their products evolve to make it easier for chemists to operate them in a way that satisfies the requirements for presentation *and* digital interpretability.

The need to improve the quality of public data, which is growing in volume at a very fast pace, is an urgent action item for the cheminformatics community, but the introduction of open lab notebooks is an opportunity to make a profound change, because unlike most other sources, the data is produced by the scientists who conduct the experiments. This immediacy removes the most intractable problems with correct data representation. That being said, if we miss this opportunity to train scientists to produce machine readable data, or fail to deliver adequate tools form to do so without an unreasonable amount of extra effort, we will end up in the unenviable position of having an ever increasing quantity of *bad* data.

Should we be successful in rising to this challenge, the outlook for cheminformatics is exciting, since this relatively young industry was incubated during a regime of scarce data, then came of age in an era of very noisy and low quality data. It is hard to know for sure how many of the common techniques in our industry provide chemical intelligence of middling quality, simply because the available training data is so poor, and requires so much effort to extract information from inappropriate data structures. As the available data simultaneously becomes more open, more abundant *and* of better quality, we can expect to see improvements to all kinds of chemical algorithms, and new use cases that were previously not viable due to data problems. We can also expect more democratisation of chemical data, since the combination of micropublications with digitally coherent content means that experimental results will often be published regardless of whether they are suitable for inclusion in a full length research article, and it also means that this data will actually be *used*. As long as the provenance of the data is retained, the data collation services that are exposed to any particular source can make their own decisions about level of trust. This is in contrast to the current situation, which more often than not can be described as blind.

The combination of these trends with use of publicly accessible social networks, such as Twitter, already has some proof of concept technology, such as the Open Drug Discovery Teams project. We anticipate that aggregation and evaluation of quality will become a highly active area of research unto itself, likely with a large crowd-sourcing component.

In this article we have concentrated primarily on chemical structures, since these are most urgently in need of attention in the field of cheminformatics, but there are numerous other kinds of metadata that can and should be incorporated into digital research publications. Allowing for different kinds of provenance is an important consideration, especially when integrating with the current open data options, e.g. whether a fact was directly provided as the result of an experiment carried out by a particular scientist, reentered from another source, text-mined from an earlier document, etc. For physical properties and activity determinations, it is useful to know more than just the units and standard errors: information about the experiment setup, calibration, the target organism, which measurement run the results were obtained from, etc., are all important. The emergence of standards for capturing this kind of high level metadata in a semantic form [66] is an essential step toward enabling the construction of algorithms that can mine the Internet for available knowledge, and create robust models that are based on something other than noise.

In short, the solution to the problem of open notebook science data quality is to apply the same level of rigour to the machine readability of the data as would normally be applied to a printable manuscript. A published paper is not considered viable until it can be understood unambiguously by chemists, and so exported digital content should not be released until a machine algorithm can interpret it without loss or corruption of essential information. Accomplishing this goal begins with the improvement of software tools for data entry and use of the most rigorously complete and well defined data formats, and culminates in changes to the culture of data publication. This culture shift requires a recognition of the primacy of machine readability: database maintainers and journals must do their best to ensure that digital content makes sense (e.g. chemical structures can be resolved to a distinct molecular formula, properties have units, etc.). The experimentalists who submit this content must be provided with better tools for avoiding common mistakes (e.g. segregating sketcher tools for creating non-chemical objects like free text or circles), and have an increased awareness of the importance of doing so. In the event of errors in digital content, the traceability of open lab notebooks leads back to the experimentalist who created it, and it must be understood that releasing flawed digital content is as much of a scientific faux pas as publishing an incorrect or misleading figure.

As cheminformaticians, these issues are our domain: it is up to us to build the tools, and ensure that they are understood and used correctly by experimentalists, so that we can leverage the full potential of open science.

## Endnotes

^a^The number of sellers and resellers of chemical compounds who make their catalogs available to download in an accessible format, such as MDL SDfile, is large. Specific instances are not listed in this article for timeliness purposes, since additions and deletions are frequent.

^b^It should be noted for completeness though that InChI does include an AuxInfo layer which can optionally encode the coordinates for a structure (http://www.inchi-trust.org/technical-faq/#11.1) but few are aware of this capability and it is rarely used.
